# From Diabetes to Oncology: Glucagon-like Peptide-1 (GLP-1) Receptor Agonist’s Dual Role in Prostate Cancer

**DOI:** 10.3390/cancers16081538

**Published:** 2024-04-18

**Authors:** Abdulrahman Alhajahjeh, Raad Al-Faouri, Hisham F. Bahmad, Taima’ Bader, Ryan W. Dobbs, Ahmed A. Abdulelah, Wassim Abou-Kheir, Elai Davicioni, David I. Lee, Mohammed Shahait

**Affiliations:** 1 School of Medicine, The University of Jordan, Amman 11190, Jordan; alhjahja2000@gmail.com; 2 King Hussein Cancer Center (KHCC), Internal Medicine Department, Amman 11190, Jordan; tym0183830@ju.edu.jo; 3 Beth Israel Deaconess Medical Center, Harvard Medical School, Boston, MA 02120, USA; ralfaour@bidmc.harvard.edu; 4 Arkadi M. Rywlin Department of Pathology and Laboratory Medicine, Mount Sinai Medical Center, Miami Beach, FL 33140, USA; hfbahmad@gmail.com; 5 Cook County Health and Hospitals System, Chicago, IL 60612, USA; ryan.dobbs@cookcountyhhs.org; 6 Edinburgh Medical School, The University of Edinburgh, Edinburgh EH8 9YL, UK; a.a.a.abdulelah@sms.ed.ac.uk; 7 Department of Anatomy, Cell Biology and Physiological Sciences, Faculty of Medicine, American University of Beirut, Beirut 1107, Lebanon; wa12@aub.edu.lb; 8 Veracyte, Inc., San Francisco, CA 94080, USA; elai.davicioni@veracyte.com; 9 Department of Urology, University of California, Irvine, CA 92868, USA; dilee1@hs.uci.edu; 10 School of Medicine, University of Sharjah, Sharjah P.O. Box 27272, United Arab Emirates

**Keywords:** prostate cancer, antidiabetic, GLP-1 receptor, GLP-1-RA, signaling pathway

## Abstract

**Simple Summary:**

In this review paper, we outline the role of GLP-1-RAs in potentially influencing PCa development and progression. While GLP-1-RAs show promise in inhibiting cancer cell proliferation, particularly when combined with metformin or radiotherapy, clinical data on their impact on PCa outcomes are limited. Future research should focus on dedicated trials to evaluate GLP-1-RA’s role in PCa management, including its potential as a prophylactic treatment and adjunct therapy in various PCa stages.

**Abstract:**

Glucagon-like peptide-1 (GLP-1), an incretin hormone renowned for its role in post-meal blood sugar regulation and glucose-dependent insulin secretion, has gained attention as a novel treatment for diabetes through GLP-1 receptor agonists (GLP-1-RA). Despite their efficacy, concerns have been raised regarding the potential associations between GLP-1-RA and certain malignancies, including medullary thyroid cancer. However, evidence of its association with prostate cancer (PCa) remains inconclusive. This review delves into the intricate relationship between GLP-1-RA and PCa, exploring the mechanisms through which GLP-1-Rs may impact PCa cells. We discuss the potential pathways involving cAMP, ERK, AMPK, mTOR, and P27. Furthermore, we underscore the imperative for additional research to elucidate the impact of GLP-1-RA treatment on PCa progression, patient outcomes, and potential interactions with existing therapies. Translational studies and clinical trials are crucial for a comprehensive understanding of the role of GLP-1-RA in PCa management.

## 1. Introduction

Glucagon-like peptide-1 (GLP-1), an incretin hormone synthesized by specialized cells in the brainstem, pancreas, and intestinal tract, plays a pivotal role in postprandial blood sugar regulation [1]. Its capacity to augment insulin release and enhance insulin’s effectiveness in managing hyperglycemia has prompted extensive research into its potential application for diabetes mellitus (DM) treatment [1,2,3,4]. However, the short half-life of GLP-1 poses a significant challenge, hindering its administration as an oral or intravenous supplement [5,6]. To overcome this limitation, GLP-1 receptor agonists (GLP-1-RAs) have been developed, addressing the pharmacokinetic hurdle associated with the hormone’s short half-life [7]. While resolving this challenge, the use of GLP-1 treatments, such as Liraglutide and Exendin-4 (Exenatide; Ex-4), has garnered attention for their potential role in weight loss management [8].

However, this therapeutic approach has not been without concerns. GLP-1-RAs have been linked to a wide range of side effects, and recent investigations have raised alarms about their potential link to thyroid cancer. A recent case–control study involving 2562 patients revealed that exposure to GLP-1-RAs for 1–3 years is associated with an increased risk of thyroid cancer (hazard ratio (HR) 1.58, 95% CI 1.27–1.95), and specifically medullary thyroid cancer (HR 1.78, 95% CI 1.04–3.05) [9,10]. While the relationship between GLP-1-RAs and other cancers, including pancreatic cancer, remains inconclusive, the evidence regarding prostate cancer (PCa) is currently insufficient [9]. Despite the higher expression of GLP-1 receptor (GLP-1-R) in ALVA-41 PCa cancer cell lines [11], the precise mechanism by which GLP-1 affects PCa cells remains understudied.

This review aims to investigate and summarize the current understanding of the possible mechanisms through which the GLP-1-R influences PCa cells. Moreover, it seeks to analyze existing clinical evidence regarding the impact of GLP-1-RA treatment on the incidence and progression of PCa in patients. Finally, we propose future directions to guide translational studies and clinical trials, focusing on the role of GLP-1-RAs in PCa management.

## 2. GLP-1 Receptor: Influences on Cellular Proliferation, Apoptosis, and Angiogenesis

GLP-1 is recognized for its role as a growth factor, exerting a compounded effect by enhancing cellular proliferation and survival. The promotion of cellular proliferation involves several pathways [12], such as the activation of the PI3K/Akt pathway, which subsequently influences the levels of pancreatic duodenal homeobox 1 (PDX1) and Forkhead box O1 (FoxO1) transcription factors [13]. In particular, GLP-1 has been observed to inhibit FoxO1 transcription factor activity, promoting β-cell proliferation. Additionally, GLP-1 has a collective impact on PDX1 by enhancing its activity and expression, contributing to the stimulation of cellular proliferation [13,14]. Nevertheless, variations in the post-transcriptional processes or alternative splicing of mRNA in different cell types may result in differential effects across various body tissues [15].

Numerous studies investigating GLP-1-RAs have demonstrated their potential to interfere with the proliferation of different cancer types, including breast, prostate, ovarian, and pancreatic cancers [16,17]. For instance, exposure of cervical cancer cells expressing proteasome alpha 2 subunit (PSMA2) to Ex-4 under high glucose conditions led to a significant reduction in tumor volume, highlighting the connection between hyperglycemia and cancer cell proliferation [18]. Additionally, positive correlations between PSMA2 and GLP-1-R expression underscore the intricate connection between these factors [18]. 

In ovarian cancer, exposure to Ex-4 has shown significant inhibition of cancer cell migration and invasion, along with enhanced apoptosis through the PI3K/AKT signaling pathway [17,19]. Gliomas, on the other hand, exhibit a biological response to Ex-4 through the GLP-1-R/Sirtuin 3 (SIRT3) signaling pathway, impeding proliferation, survival, and migration [20]. In endometrial cancer, Ex-4 induces apoptosis by activating the AMP-activated protein kinase (AMPK) signaling pathway [21]. However, in pancreatic cells, liraglutide hampers the AKT and extracellular-signal-regulated kinase 1/2 (ERK1/2) signaling pathways, leading to apoptosis and inhibition of cell proliferation in vivo [22]. 

GLP-1 also plays a crucial role in modulating cellular apoptosis, exerting prominent direct anti-apoptotic effects via a multitude of pathway activations, which vary according to the specific tissue [23]. GLP-1 enhances the ATP/AMP ratio, inducing apoptosis via activation of AMPK in beta-pancreatic cells [24,25]. It also activates the protein kinase A (PKA)-dependent pathway, exhibiting profound activation of pancreatic duodenal homeobox 1 (PDX-1) [26]. In cardiomyocytes, GLP-1 activates the PI3K/Akt pathway to suppress apoptosis [25,27]. Furthermore, GLP-1 inhibits caspase-3, a pivotal player in apoptosis [25]. 

GLP-1 has been postulated to exert a pro-angiogenic effect, as demonstrated by Aronis et al., who employed an in vitro model utilizing human umbilical vein endothelial cells (HUVECs). Their study showcased the dose-dependent impact of GLP-1 on prompting and stimulating angiogenesis [28]. The initiation of this angiogenic effect involves the activation of multiple pathways, including PI3K/Akt, Src, and PKA. Confirming the pro-angiogenic effects of GLP-1, the introduction of inhibitors to these pathways resulted in a marked reduction in angiogenesis [28]. 

It is important to note that the influence of GLP-1-RAs extends beyond a direct impact on cells. These agents indirectly contribute to reducing insulin levels, aiding in the treatment of insulin resistance within the body [29]. Insulin, functioning as a growth hormone by binding to the insulin-like growth factor receptor (IGF-R), has been linked to a more aggressive course of cancer in patients with type 2 diabetes mellitus (T2DM), as indicated by prior studies [30].

## 3. GLP-1 Receptor: Modulating Insulin Resistance, Inflammation, and Cancer Development

GLP-1 has demonstrated its capability to inhibit inflammatory processes, especially those associated with macrophages, such as the NF-κB pathway. Guo et al. conducted a study utilizing GLP-1-RAs to assess the anti-inflammatory characteristics of GLP-1 [31]. Their findings revealed a marked reduction in macrophage infiltration and suppression of cytokine release, including TNF-β, IL-6, and IL-1β, in the macrophage cell line (RAW264) exposed to Ex-4 in vivo [31]. These cytokines play a pivotal role in chronic inflammatory processes implicated in the development of insulin resistance [32]. 

Moreover, a body of evidence has established an association between inflammation and an elevated incidence of PCa, whether attributable to bacterial or viral infections or of unknown etiology. A 2014 meta-analysis unveiled a 49% higher incidence of PCa in patients with a history of sexually transmitted diseases. Additionally, infections caused by the BK virus and human papillomavirus (HPV) have also been implicated [33,34,35]. Notably, histopathological inflammation, even in the absence of an identified organism, correlates with the prevalence of PCa and high-grade disease [36,37]. Inflammation induces remodeling of the extracellular matrix and triggers epithelial-to-mesenchymal transition in PCa [38]. 

In the context of GLP-1-RAs, it is hypothesized that their anti-inflammatory effects contribute to a reduction in and potentially complete inhibition of insulin resistance [31]. Considering the increased risk of malignancy in patients with insulin resistance, largely attributed to elevated production of reactive oxygen species influencing carcinogenesis and mutagenesis, GLP-1 is believed to influence the development of malignancy by impeding this pathological pathway [39].

## 4. Impact of GLP-1 Receptor Agonists on Prostate Cancer Cells: Mechanisms and Signaling Pathways

The investigation into GLP-1-R action mechanisms and signaling pathways in cancer cells has been a focal point for numerous researchers. The GLP-1-R, classified as a G-protein-coupled receptor, triggers the activation of adenylyl cyclase, leading to an elevation of intracellular cAMP levels [40]. The cAMP cascade involves two primary pathways. Firstly, it inhibits the action of extracellular-signal-regulated kinases (ERK), a pivotal activator of Cyclin D1 [41]. Cyclin D1, in turn, drives the phosphorylation of the retinoblastoma protein, impeding its binding to DNA, thus hindering DNA replication and the initiation of the S phase of the cell cycle. A study by Nomiyama et al. demonstrated that Ex-4 decreases ERK-MAPK phosphorylation in LNCap PCa cells [41]. Furthermore, inhibiting ERK leads to cell cycle arrest at the G1/S phase junction [41]. Simultaneously, cAMP activation triggers phosphokinase A, subsequently inducing AMPK [42]. AMPK then counteracts mTOR, a central controller influencing cellular activities, including nucleotide, ribosome, protein, and lipid synthesis, as well as autophagy suppression [43,44,45]. P27, a critical antiproliferative protein in PCa, is significantly affected by these processes [46]. It inhibits cyclin-dependent kinase 2 (CDK2), a key cell growth promoter managing the transition from the G1/S phase to the G2/M phase [11,47]. Additionally, P27 obstructs RhoA signaling, a critical pathway that controls cancer cell movement and tissue infiltration [48].

It is worth mentioning that the utilization of two different GLP-1-RAs has revealed an alternative pathway related to the activation of p38-MAPK. This is evidenced by the increase in the level of phosphorylated P38, a major regulator in cancers with varying roles depending on the cell type [42].

**Figure 1:**
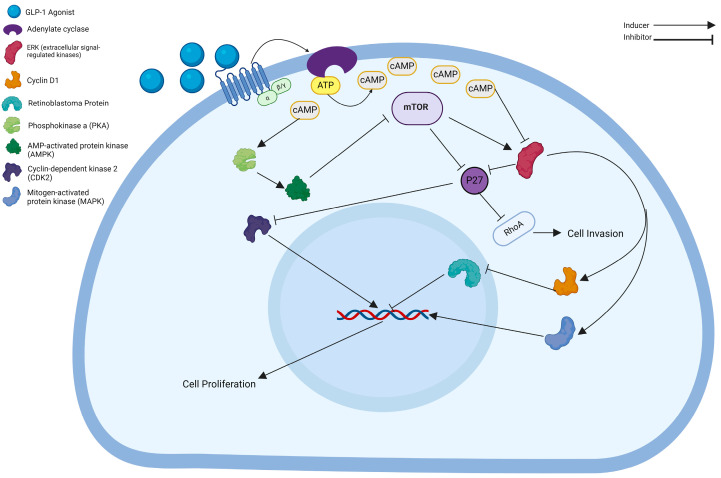
The figure illustrates the cellular pathways of the effect of GLP-1-RA inside prostate cancer (PCa) cells: GLP-1-R is a G-protein-coupled receptor activating Adenylyl cyclase, leading to elevated cAMP levels. The cAMP cascade inhibits ERK, suppressing Cyclin D1 activation and preventing DNA replication. Simultaneously, cAMP activates PKA, prompting AMPK, counteracting mTOR, and influencing various cellular processes. P27, an antiproliferative protein, restrains CDK2, inhibiting cell cycle progression. In prostate cancer, P27 obstructs RhoA signaling, impacting cell migration. Additionally, GLP-1-RA inhibits cell proliferation and tissue invasion by increasing cAMP levels and indirectly through P27. The pathway also involves p38-MAPK activation, showing cell-type-dependent effects. This intricate network provides insights into the potential therapeutic impact of GLP-1-RA in prostate cancer. Created with BioRender.com (accessed on 20 March 2024).

P27 inhibits mTOR through the mediation of S-phase kinase-associated protein 2 (SKP2) degradation and ESRK. Consequently, GLP-1-RAs directly impede both cell proliferation and tissue invasion by increasing the intracellular levels of cAMP through P32. A summary of these pathways is illustrated in Figure 1.

Androgen deprivation therapy (ADT) stands as one of the mainstay treatments for advanced PCa. Wenjing et al. underscored that Ex-4 reduces resistance to androgen receptor (AR) inhibitors, showcasing its impact on the PI3K/AKT/mTOR pathway [49]. 

It is noteworthy to mention that clonal selection of cancer cells for treatment with GLP-1-RAs is affected by the expression of GLP-1-Rs in those cells. A study by Nomiyama et al., performed on the cell lines LNCap and ALVA-41 (androgen-sensitive cell lines) and PC3 and DU145 (androgen-independent cell lines), demonstrated that GLP-1-R mRNA was abundantly expressed in LNCap and DU145 cells, but was significantly lower in PC3 and ALVA-41 cells [41].

## 5. GLP-1 Receptor Agonists and Their Impact on Prostate Cancer Cells: In Vitro Insights

The mechanisms of action of GLP-1-Rs and their signaling pathways in PCa cells have been studied in a variety of settings (Table 1 and Table 2). Initially, the hypothesis that the activation of GLP-1-Rs was linked to the suppression of androgen hormone activity within cells underwent scrutiny in various studies. Contrary to the initial hypothesis, these studies demonstrated no discernible alteration in AR expression within prostate cells during GLP-1-RA treatment [41].

Several investigations have explored the effect of GLP-1-RA on PCa growth by inoculating specific PCa cell types into mice [11,41,42,50]. Nomiyama et al. illustrated that treatment of PCa cell lines with Ex-4 resulted in a reduction of over 50% in PCa cell volume for both low- and high-Ex-4-dose groups compared to the control group [41]. In vivo experiments in this study revealed a substantial decrease in the fraction of P405S-, Ki97-, and pERK-MAPK-positive cells, with a low Ex-4 dose showing a reduction of approximately 50% and a roughly 25% reduction with a high Ex-4 dose [41]. While a decline in blood prostate-specific antigen (PSA) levels was observed across groups, statistical significance was not achieved in the comparison of the control vs. low dose (*p* = 0.11) and control vs. high dose (*p* = 0.08) groups, possibly attributed to the relatively small sample size [41].

In animal models simulating advanced stages of PCa, combining Ex-4 with radiotherapy yielded significantly greater reductions in tumor size than either treatment alone. The synergistic effect was notable, with a 64 ± 6.7% reduction for the Ex-4 and radiotherapy combination compared to 30.1 ± 9.0% and 50.3 ± 6.4% for Ex-4 and radiotherapy separately [42]. This synergy is attributed to the dual impact on G2/M cell cycle arrest, with Ex-4 additionally inducing G1/S cell cycle arrest, enhancing the efficacy of radiotherapy [42]. Furthermore, when combining GLP-1-RA with Docetaxel, a significant arrest of the cell cycle at the G2/M phase was observed compared to Docetaxel or GLP-1-RA alone, along with increased apoptotic genes such as BAX gene expression and decreased expression of anti-apoptotic genes such as BCL2 [51].

Regarding the possible side effects of GLP-1-RA in mice with PCa, the results have been mixed regarding increased insulin levels and weight gain [41,42,50,51]. Some studies found no noticeable changes, while others observed an increase in weight and insulin levels with GLP-1-RA. Interestingly, a study assessing the effect of combined treatment with Ex-4 and Metformin on PCa cell growth highlighted that the group of mice administered the combined treatment showed decreased insulin levels compared to the control level, suggesting a potential augmentation of the effect of GLP-1-RA by Metformin [50]. However, this effect was not observed when combining GLP-1-RA with radiotherapy [42]. These studies underscore the potential of GLP-1-RA, particularly Ex-4, to mitigate PCa progression. Combining Ex-4 with Metformin or radiotherapy demonstrated compelling synergistic effects, warranting further investigation in randomized clinical trials.

A possible mechanism of the interaction of GLP-1-RA with other diabetic agents, including Metformin, could be via activating AMPK, a signaling pathway that works with GLP-1-RA to inhibit cell growth and migration [7,50]. Remarkably, when GLP-1-RA and Metformin were administered together to treat PCa cells, a significant reduction in PCa tumor volume was observed, surpassing the effects of a placebo or the drug alone [50]. Their combined action exhibited a synergistic effect, increasing the potential therapeutic impact.

It was observed that a higher expression of GLP-1-Rs was found in patients with low-Gleason-score tumors compared to patients with high Gleason scores [11]. While in vitro assessments of these drugs have shown promising outcomes, their role in inducing apoptosis within PCa cells remains elusive [42,50].

## 6. Prostate Cancer and Metabolic Syndrome

In 1988, Reavan introduced Syndrome X, a complex condition characterized by several components, including resistance to insulin-stimulated glucose uptake, glucose intolerance, hyperinsulinemia, increased very-low-density lipoprotein, elevated triglyceride levels, reduced high-density lipoprotein (HDL), and hypertension [53]. In the modern era, this condition is recognized as metabolic syndrome, a constellation of risk factors for T2DM and cardiovascular diseases [54]. Nevertheless, the impact of metabolic syndrome extends beyond these health concerns, affecting other systems and organs, including the central nervous system, kidneys, lungs, and various cancers [54]. Results from a recent meta-analysis shed light on the increased incidence of PCa among individuals with metabolic syndrome, highlighting an odds ratio (OR) of 1.17 (95% CI: 1.00–1.36, *p* = 0.04) [55]. Furthermore, this study demonstrated an association between metabolic syndrome and higher-grade tumors, with an OR of 1.89 (95% CI: 1.50–2.38, *p* < 0.0001) [55]. The observed correlation between metabolic syndrome and elevated cancer risk may be attributed to heightened insulin levels, recognized as growth hormones, and implicated in the development of various cancer types, including PCa [39,53]. This finding emphasizes the intricate relationship between metabolic health and prostate cancer risk.

Studies have illuminated the association between metabolic syndrome and adverse outcomes in patients with advanced prostate cancer (PCa), revealing shorter progression-free survival (PFS) and overall survival (OS). For example, in a study involving 551 metastatic castration-resistant PCa patients, the median PFS for those with metabolic syndrome was 3.7 months compared to 8.7 months for patients without metabolic syndrome, with an HR of 2.77 (95% CI 2.12–3.61; *p* < 0.0001) [56]. Similarly, the median OS for patients with metabolic syndrome was 6.9 months, whereas patients without metabolic syndrome survived for 19 months, yielding an HR of 3.43 (95% CI 2.56–4.58; *p* < 0.0001) [56]. This substantial difference indicates a notable gap of 7.5 years versus 10.6 years in favor of patients without metabolic syndrome [56].

Furthermore, there is an increased risk of advanced-clinical-stage PCa in patients with metabolic syndrome (OR: 2.23; 95% CI: 1.273–3.893) [57]. Patients with metabolic syndrome also face a higher risk of PCa-specific mortality, with a relative risk (RR) of 1.12 (95% CI: 1.02–1.23) [58]. Although body mass index (BMI) lacks a clear association with OS in PCa patients, elevated metabolic gene expression, particularly prevalent in patients with metabolic syndrome, is linked to unfavorable outcomes [59]. Additionally, having more components of metabolic syndrome is associated with higher-grade PCa and poorer outcomes. Patients with three or more components of metabolic syndrome exhibit a higher risk of PCa (OR: 1.54; 95% CI: 1.17–2.04; *p* = 0.002), castrate-sensitive PCa (OR: 1.56; 95% CI: 1.17–2.08; *p* = 0.002), and intermediate- to high-grade PCa (OR: 1.56; 95% CI: 1.16–2.10; *p* = 0.003) [59]. Regarding the outcomes of radical prostatectomy for non-metastatic PCa, metabolic syndrome is associated with a less favorable prognosis, and each component of metabolic syndrome independently predicts adverse patient outcomes [57].

The relationship between T2DM and PCa is not unidirectional. ADT targets the effects of androgens in the body and inhibits their impact on PCa cells [60]. One of the side effects of ADT, as shown by a recent meta-analysis, is that it increases the risk of developing T2DM by 43% and hypertension by 30%, which might increase the risk of metabolic syndrome, but the association did not reach statistical significance [61]. 

Recognizing the importance of treating metabolic syndrome and DM to improve patient outcomes, antidiabetic medications, particularly thiazolidinediones and GLP-1-RAs, have shown promise. They are associated with a reduced risk of developing PCa, with OR of 0.55 (*p* = 0.04) and 0.53 (*p* = 0.006), respectively, as indicated by a recent meta-analysis [62]. However, the impact of these medications on the progression and outcomes of PCa patients remains an area requiring further investigation, particularly with the growing adoption of GLP-1-RA treatments. 

## 7. Current Clinical Outcomes of GLP-1-RAs in Prostate Cancer Patients

A meta-analysis by Cui et al., encompassing 47 studies and 3,094,152 diabetes patients, revealed no significant association between the use of Metformin, thiazolidinediones, sulfonylureas, insulin, or dipeptidyl peptidase-4 inhibitors and the risk of PCa (all *p*-values > 0.05) [62]. However, in randomized controlled trials (RCTs), a notable decrease in the risk of PCa was observed with thiazolidinediones (OR: 0.55; *p* = 0.04) and GLP-1-RA (OR: 0.53; *p* = 0.006), while no significant correlation was found with SGLT2 inhibitors (*p* = 0.3) [62].

Moreover, an inverse relationship between the proportion of GLP-1-R in PCa cells identified through biopsies and patient prognosis was noted [11]. Nevertheless, comprehensive data addressing the influence of GLP-1-RA use on disease progression and OS are lacking, hindered by the absence of dedicated clinical trials and extensive retrospective multicenter investigations. Gaps in the literature persist regarding interactions with other treatments, overall patient well-being, cost-effectiveness, and the intricate mechanisms underlying the effect on cancer cells. 

## 8. Future Directions

It is worth exploring the potential benefits of GLP-1-RA as a prophylactic treatment in individuals with metabolic syndrome and a high risk of developing PCa, such as those with a strong family history of lethal PCa or who are carriers of *BRCA* genes.

Guidelines from major urology associations have emphasized the role of active surveillance (AS) in the management of low-risk PCa [63]. These recommendations have led AS to become the preferred initial management approach for low-risk PCa, as observed in several contemporary registries [63]. However, one-third of patients with AS end up receiving definitive treatment for disease progression or anxiety related to AS, and more men are seeking focal therapy for intermediate-risk disease [64,65]. As the evidence shows high expression of GLP-1-R with low Gleason grade, there is potential for using GLP-1-RA as an adjunct treatment to prevent disease progression, subsequently decreasing the dropout from AS and delaying the need for definitive PCa treatment. 

In addition, GLP-1-RA might need to be explored in the context of focal therapy as an adjunct treatment or adjuvant treatment after radical prostatectomy and definitive radiation in patients at increased risk of biochemical recurrence. Furthermore, GLP-1-RA might be investigated as an adjunct treatment for metastatic hormone-sensitive disease to reverse insulin resistance induced by ADT. It is worth exploring the ability of GLP-1-RA treatment to overcome AR resistance observed in castration-resistant PCa. Furthermore, clonal selection of cancer cells for treatment with GLP-1-RAs is essential, and this affects disease progression, especially because PCa cells demonstrate differential expression of these receptors [41].

## 9. Conclusions

In summary, while preclinical studies have shown promising results, with GLP-1-RA inhibiting cancer cell proliferation and showing synergistic effects when combined with Metformin or RT, clinical data on its impact on PCa outcomes are currently insufficient. This review highlights the need for dedicated trials to assess GLP-1-RA’s role in disease progression and patient survival, as well as its potential interactions with other treatments. Exploring GLP-1-RA in various PCa stages, including AS, presents promising avenues for future research.

## Figures and Tables

**Table 1:** Table summarizing the in vivo tumor response of GLP-1-RAs on various PCa cells.

Author	Cell Linage	Drug	Tumor Response
Nomiyama T. et al. (2014) [41]	LNCap human androgen-sensitive PCa cell line, and the PC3 and DU145 human androgen-independent PCa cell.	Ex-4	Tumor volume: after 12 weeks Control group: around 550 mm^3^ Low dose Ex-4 = around 200 mm^3^ High dose Ex-4 = around 200 mm^3^
He W. et al. (2018) [42]	LNCap, Du145, PC3, ArCaP, and ALVA-41	Ex-4 and IR	Tumor volume: after 4 weeks Control: around 1850 mm^3^ IR: around 900 mm^3^ Ex-4: around 1400 mm^3^ IR + Ex-4: around 500 mm^3^
Shigeoka T. et al. (2020) [11]	ALVA-41 and ALVA-41 cells induced GLP-1-R	Ex-4	Tumor volume: after NA ALVA-41 without Ex-4: around 2200 mm^3^ ALVA-41 with Ex-4: around 1500 mm^3^ ALVA-41 induced GLP-1-R without Ex-4: around 1000 mm^3^ ALVA-41 induced GLP-1-R with Ex-4: around 600 mm^3^
Tsutsumi Y. et al. (2015) [50]	LNCaP, PC3, and DU145 cells	Ex-4 and Metformin	Tumor volume: after 12 weeks Control: around 490 mm^3^ Ex-4: around 190 mm^3^ Metformin: around 180 mm^3^ Ex-4 + Metformin: around 85 mm^3^
Eftekhari S. et al. (2020) [51]	LNCaP	Liraglutide and Docetaxel	Tumor volume: no data % of apoptotic cells: Control: around 5% Liraglutide: around 7% Docetaxel: around 12% * Docetaxel + Liraglutide: around 17% **

Abbreviations: Ex-4: exenatide-4; IR: intervention radiology; * statistically significant; ** highly statistically significant.

**Table 2:** Table summarizing the mechanisms of action of GLP-1-RAs.

Author	Drug	Explanation
Eftekhari S. et al. (2020) [51]	Liraglutide and Docetaxel	Significant arrest of G2/M with significant decrease in BCL-2 mRNA and increase in BAX expression shown when both treatments were combined compared to when they were used alone. Docetaxel with Liraglutide showed a significant increase in the phosphorylation of ERK1/2 and AKT compared to other groups.
Tsutsumi Y. et al. (2015) [50]	Exendin–4 and Metformin	P504S dramatically decreased with Ex-4, Metformin, and the combined treatment. No change was observed in AR expression after Ex-4 and Metformin treatment in the PCa tumor. Ex-4 and Metformin treatment alone for 24 h significantly decreased DNA synthesis.
Shigeoka T. et al. (2020) [11]	Exendin–4 and Metformin	Ex-4 slowed cell growth by using two pathways, GLP-1-R and cAMP-protein kinase A. It made the ERK pathway slower, reducing proteins that fuel cell division. It boosted P27, a cell regulator, by inhibiting protein from the SKP2 gene and decreased the expression of the gene, ultimately inhibiting conversion from the Phase G1-S phase.
Li X.N. et al. (2017) [52]	Exendin and Liraglutide	Exenatide concentration was associated with an increase in prostate cancer cell apoptosis due to an increase in the ratio of Bax to Bcl-2 proteins in a dose-dependent manner. Regarding the liraglutide, there was no statistical difference in apoptosis between the control group and the 1 nmol/L liraglutide group, with no significant difference between the 10 nmol/L and 100 nmol/L liraglutide groups. Treatment activated a signaling pathway called p38-MAPK, as indicated by an increase in the ratio of phosphor-p38 to total p38.
He W. et al. (2018) [42]	Exendin	Ex-4 increased AMPK phosphorylation and decreased the levels of p-mTOR and cyclin B, which inhibit the proliferation.
Nomiyama T. et al. [41]	Ex-4	Increased cAMP led to decreased extracellular-signal-regulated kinase (ERK) and mitogen-activated protein kinase (MAPK) phosphorylation in PCa cells.
Wenjing H. et al. (2020) [49]	Exendin-4 enhances enzalutamide	The combination of treatments significantly reduced Akt and mTOR levels, which were triggered by enzalutamide administration.
